# The Triglycerides, total Cholesterol, and Body weight Index associating with frailty and predicting poor outcome after transcatheter aortic valve implantation: insights from LAPLACE-TAVI registry

**DOI:** 10.1093/ehjopen/oeaf008

**Published:** 2025-01-28

**Authors:** Shinichiro Doi, Takehiro Funamizu, Hiroshi Iwata, Ryo Naito, Soshi Moriya, Takuma Koike, Ryota Nishio, Norihito Takahashi, Yuichi Chikata, Seiji Koga, Shinya Okazaki, Ryosuke Higuchi, Itaru Takamisawa, Mike Saji, Kei Sato, Harutoshi Tamura, Hiroaki Yokoyama, Takayuki Onishi, Tetsuya Tobaru, Shuichiro Takanashi, Minoru Tabata, Tohru Minamino

**Affiliations:** Department of Cardiovascular Biology and Medicine, Juntendo University Graduate School of Medicine, 2-1-1 Hongo, Bunkyo-ku, Tokyo 113-8421, Japan; Department of Cardiovascular Biology and Medicine, Juntendo University Graduate School of Medicine, 2-1-1 Hongo, Bunkyo-ku, Tokyo 113-8421, Japan; Department of Cardiovascular Biology and Medicine, Juntendo University Graduate School of Medicine, 2-1-1 Hongo, Bunkyo-ku, Tokyo 113-8421, Japan; Department of Cardiovascular Biology and Medicine, Juntendo University Graduate School of Medicine, 2-1-1 Hongo, Bunkyo-ku, Tokyo 113-8421, Japan; Department of Cardiovascular Biology and Medicine, Juntendo University Graduate School of Medicine, 2-1-1 Hongo, Bunkyo-ku, Tokyo 113-8421, Japan; Department of Cardiovascular Biology and Medicine, Juntendo University Graduate School of Medicine, 2-1-1 Hongo, Bunkyo-ku, Tokyo 113-8421, Japan; Department of Cardiovascular Biology and Medicine, Juntendo University Graduate School of Medicine, 2-1-1 Hongo, Bunkyo-ku, Tokyo 113-8421, Japan; Department of Cardiovascular Biology and Medicine, Juntendo University Graduate School of Medicine, 2-1-1 Hongo, Bunkyo-ku, Tokyo 113-8421, Japan; Department of Cardiovascular Biology and Medicine, Juntendo University Graduate School of Medicine, 2-1-1 Hongo, Bunkyo-ku, Tokyo 113-8421, Japan; Department of Cardiovascular Biology and Medicine, Juntendo University Graduate School of Medicine, 2-1-1 Hongo, Bunkyo-ku, Tokyo 113-8421, Japan; Department of Cardiovascular Biology and Medicine, Juntendo University Graduate School of Medicine, 2-1-1 Hongo, Bunkyo-ku, Tokyo 113-8421, Japan; Department of Cardiology, Sakakibara Heart Institute, 3-16-1 Asahicho, Fuchu, Tokyo 183-0003, Japan; Department of Cardiology, Sakakibara Heart Institute, 3-16-1 Asahicho, Fuchu, Tokyo 183-0003, Japan; Division of Cardiovascular Medicine, Department of Internal Medicine, Faculty of Medicine, Toho University, 6-11-1 Omori-nishi, Ota-ku, Tokyo 143-8541, Japan; Department of Cardiology and Nephrology, Mie University Graduate School of Medicine, 2-174 Edobashi, Tsu, Mie 514-8507, Japan; Department of Cardiology, Pulmonology and Nephrology, Yamagata University Hospital, 2-2-2 Iida-Nishi, Yamagata 990-9585, Japan; Department of Cardiology and Nephrology, Hirosaki University Graduate School of Medicine, 5 Zaifu-cho, Hirosaki 036-8562, Japan; Department of Cardiology, Kawasaki Saiwai Hospital, 31-27 Omiya, Saiwai, Kawasaki, Kanagawa 212-0014, Japan; Department of Cardiology, Kawasaki Saiwai Hospital, 31-27 Omiya, Saiwai, Kawasaki, Kanagawa 212-0014, Japan; Department of Cardiovascular Surgery, Kawasaki Saiwai Hospital, 31-27 Omiya, Saiwai, Kawasaki, Kanagawa 212-0014, Japan; Department of Cardiovascular Surgery, Sakakibara Heart Institute, 3-16-1 Asahicho, Fuchu, Tokyo 183-0003, Japan; Department of Cardiovascular Surgery, Juntendo University Graduate School of Medicine, 2-1-1 Hongo, Bunkyo-ku, Tokyo 113-8421, Japan; Department of Cardiovascular Biology and Medicine, Juntendo University Graduate School of Medicine, 2-1-1 Hongo, Bunkyo-ku, Tokyo 113-8421, Japan

**Keywords:** Transcatheter aortic valve implantation, Prognosis, Nutritional index, Frailty

## Abstract

**Aims:**

The nutritional status and frailty are crucial in patients with aortic stenosis undergoing transcatheter aortic valve implantation (TAVI), as they significantly impact outcomes. We have previously developed an easily calculable nutritional index, TCBI (Triglycerides, total Cholesterol, and Body weight Index), which has been validated as a prognostic indicator in various cardiovascular disease contexts. This study aimed to evaluate the impact of a low TCBI on the frailty and outcomes of patients undergoing TAVI.

**Methods and results:**

This study is a part of a Japanese multi-centre prospective registry database of TAVI cases (*n* = 824). Participants were categorized into three groups based on TCBI tertiles before TAVI. The primary endpoint was all-cause mortality with a follow-up duration of up to 3 years. In the lowest TCBI tertile group, motor functions reflecting frailty were substantially impaired, and cumulative incidences of primary endpoint was significantly higher compared to other groups. Multivariate Cox proportional hazard analyses adjusted by risk factors for poor outcomes following TAVI identified low TCBI significantly associated with an increased risk of the primary endpoint [hazard ratio (HR) and 95% confidence interval (95% CI) of 1 SD lower TCBI for all-cause mortality: 1.52, 1.08–2.13, *P* = 0.015]. Moreover, in individuals who experienced serious preprocedural complications, the negative prognostic impact of low TCBI was significantly amplified (HR and 95% CI: 4.9, 1.9–12.5, *P* < 0.001).

**Conclusion:**

The present findings underscore the importance of nutritional assessment in patients undergoing TAVI. TCBI proved useful for accurate risk stratification and determining TAVI procedural strategies.

## Graphical abstract

**graphical abstract:**
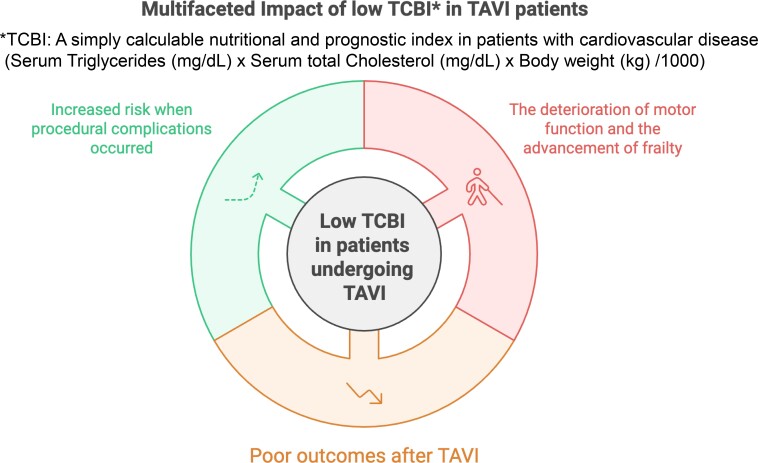

## Introduction

The prevalence of aortic stenosis (AS) is on the rise due to the progressive aging of societies.^1,2^ Transcatheter aortic valve implantation (TAVI) has emerged as an effective treatment for AS, initially developed as an alternative for patients at high risk for surgical aortic replacement (SAVR), and it is expanding to include patients at lower surgical risk.^3,4^ Given that the majority of patients undergoing TAVI are elderly, it is essential to provide them with comprehensive healthcare approaches that encompass crucial elements, such as appropriate nutrition and exercise, in addition to medical and interventional treatments.

Frailty and malnutrition are two major interrelated and frequently coexisting risk factors that significantly burden older adults with cardiovascular disease.^5^ These factors are associated with increased risks of various morbidities and even mortality. Studies in patients with AS and those undergoing SAVR or TAVI have demonstrated an association between frailty or malnutrition and an increased risk of poor outcomes, highlighting their clinical importance as prognostic factors in patients undergoing TAVI.^1^

Furthermore, low nutritional indices, such as the Geriatric Nutritional Risk Index (GNRI) and controlling nutritional status (CONUT), have proven effective as indicators of poor outcomes in patients following TAVI.^6^ In this context, we have proposed a novel nutritional index. Existing nutritional assessment tools such as GNRI and CONUT, while valuable, involve more complex calculations and multiple parameters, which can hinder their rapid application in clinical settings. In contrast, the Triglycerides, total Cholesterol, and Body weight Index (TCBI) offers a simplified approach, requiring only serum triglycerides, total cholesterol, and body weight, thereby facilitating more efficient prognostic evaluations in patients undergoing TAVI. TCBI has been shown to have a positive linear correlation with GNRI in patients with coronary artery disease (CAD),^7^ critical patients with cardiovascular disease,^8^ and those with heart failure.^9^ Additionally, other groups have also validated the utility of TCBI in patients with stroke,^10^ elderly patients with CAD,^11^ and those with heart failure.^12^

Despite these promising associations, no study has yet addressed the utility of TCBI as an indicator of nutritional status, frailty, and outcomes in patients following TAVI. Thus, the objective of this multi-centre registry-based observational study was to assess whether TCBI could serve as a valuable tool for estimating frailty and predicting prognosis in patients with AS after TAVI.

## Methods

### Study participants

This study is a retrospective observational analysis of data from the prospective multi-centre registry database involving patients who underwent TAVI at six centres in Japan, including two municipal and four university hospitals. The registry, named The aLliAnce for exPloring cLinical prospects of AortiC valvE disease (LAPLACE-TAVI registry), was utilized for this analysis. The participating institutions were the Sakakibara Heart Institute, Juntendo University Hospital, Yamagata University Hospital, Hirosaki University Hospital, Mie University Hospital, and Kawasaki Saiwai Hospital. In each participating centre, the indication for TAVI was determined by the heart team based on risk estimation.

For the present analysis, we enrolled a consecutive total of 824 patients who underwent TAVI between October 2013 and February 2020 and had available data on components for calculating TCBI [serum triglycerides (TG), serum total cholesterol (TC), and body weight (BW)] at the time of the TAVI procedure. Participants were then divided into three groups based on TCBI tertiles as follows: T1 (lowest tertile; TCBI < 645.6, *n* = 275), T2 (middle tertile; 645.6 ≤ TCBI ≤ 1069.2, *n* = 275), and T3 (highest tertile; TCBI > 1069.2, *n* = 274) (see Supplementary material online, *Figure S1*).

### Ethical considerations

This study was conducted in accordance with the Declaration of Helsinki and approval was obtained from the Institutional Review Board (IRB) of each institution; Sakakibara Heart Institute (IRB-ID: 17-048), Juntendo University Hospital (IRB-ID: 17-263), Yamagata University Hospital (IRB-ID: 2019-407), Hirosaki University Hospital (IRB-ID: 2020-040), Mie University Hospital (IRB-ID: H2021-049), and Kawasaki Saiwai Hospital (IRB-ID: R4-13). The registry was publicly registered in the University Medical Information Network Japan-Clinical Trials Registry (UMIN000031133). Written informed consent was obtained from all participants included in this registry.

### Measurements of nutritional indices (Triglycerides, total Cholesterol, and Body weight Index and Geriatric Nutritional Risk Index), 5 m walk test, and handgrip strength

The nutritional indices TCBI and GNRI were calculated using established formulas.^7,13^ TCBI was derived from the equation: TCBI = (serum triglycerides (TG, mg/dL) × serum total cholesterol (TC, mg/dL) × body weight (BW, kg))/1000, while GNRI was calculated using: GNRI = 14.89 × serum Alb (g/dL) + 41.7 × (measured body weight (kg)/ideal body weight (kg))². The Lorentz formula was used to determine ideal body weight, where ideal body weight = (height (cm) − 100) − ((height (cm) − 150)/4) for men, and (height (cm) − 100) − ((height (cm) − 150)/2) for women.

The 5 m walk test (5MWT) measured the time (in seconds) taken by patients to walk 5 m at a comfortable pace. The test was repeated three times, and the average time was recorded. Handgrip strength (kg) was measured by squeezing a handgrip dynamometer with maximum force in either hand. The test was also repeated three times, and the highest value from either hand was recorded. Both the 5MWT and handgrip strength measurements were conducted by trained physical therapists.

### Definitions of outcome measures, serious periprocedural complications of transcatheter aortic valve implantation, and follow-up duration

The primary outcome measure was all-cause mortality, while the secondary outcome measure was the composite of all-cause mortality and heart failure hospitalization. In this study, serious periprocedural complications of TAVI were defined as any of following events based on the Valve Academic Research Consortium-3 (VARC-3) criteria, including major vascular complications, cardiac structural complications, disabling stroke, conduction disturbance, valve malposition requiring reintervention, and life-threatening bleeding^14^ The follow-up duration was up to 3 years, with a median follow-up period of 1.67 years.

### Statistical analysis

Continuous variables were presented as the mean ± standard deviation or median with interquartile range, based on the results of the Shapiro––Wilk normality test. Categorical data were presented as numbers and percentages. Parametric Pearson or non-parametric Spearman correlation was used to evaluate relationships between two continuous variables as appropriate. Study participants were divided into three groups based on TCBI tertiles, defined as follows: lowest tertile (T1), mid tertile (T2), and highest tertile (T3). Quantitative data across groups were compared using ANOVA or the Kruskal–Wallis test, depending on the normality of variable distributions. Categorical variables among groups were compared using the Fisher exact test. Unadjusted Kaplan–Meier analysis was performed to evaluate the time to the cumulative incidence of primary and secondary outcome measures, followed by log-rank comparisons among groups. Multivariate Cox proportional hazard analyses were conducted using two models that incorporated variables previously identified to be associated with an elevated risk of mortality with or without heart failure hospitalization in patients following TAVI.^15,16^ For the calculation of hazard ratios of lower TCBI as a continuous variable for both primary and secondary outcome measures, Model 1 was adjusted for age and sex, while Model 2 was adjusted for age, sex, history of heart failure, and atrial fibrillation. Additionally, cubic spline curves were illustrated to show age- and sex-adjusted hazard ratios and 95% confidence intervals of TCBI for primary and secondary outcome measures, using the median TCBI value (834.3) as the reference. Statistical significance was defined as a *P* < 0.05. All analyses were performed using statistical software (JMP Pro 16.0; SAS Institute Inc., Cary, NC, USA, and IBM SPSS Statistics, Version 27.0. Armonk, NY, USA).

## Results

### Baseline background demographics, comorbidities, medications, and procedural characteristics by tertiles of Triglycerides, total Cholesterol, and Body weight Index

We categorized the study participants into three groups (group T1: TCBI < 645.6, *n* = 275, group T2: 645.6 ≤ TCBI ≤ 1069.2, *n* = 275, and T3: TCBI > 1069.2, *n* = 274). The baseline background demographics, medications, and procedural characteristics for each group are summarized in *Table 1*. Overall, patients with lower TCBI exhibited more complex background characteristics compared to those with preserved TCBI. Consistently, the surgical risk scores, including logistic EURO score and STS risk score, were significantly higher in the T1 group. Specifically, individuals in the T1 group were older and had a higher prevalence of a history of heart failure, symptoms indicative of NYHA class 3 or 4, atrial fibrillation/atrial flutter (AF/AFL), and moderate or more severe mitral and tricuspid regurgitation (MR and TR). Patients in the T1 group were more likely to be taking diuretics. In contrast, renal function was impaired in the T3 group with higher serum creatinine levels compared to the T1 and T2 groups. Procedure- and device-related parameters of TAVI, such as procedure time, approach site (transfemoral vs. other approaches), and type and size of implanted transcatheter valves (self-expandable vs. balloon-expandable), did not differ significantly among the groups. While the incidence rate of major complications was slightly higher in the T1 group, there was no statistically significant difference observed among the groups.

**Table 1:** Baseline clinical characteristics of the study population according to tertile of Triglycerides, total Cholesterol, and Body weight Index

	Overall	Lowest tertile group (T1)	Mid tertile group (T2)	Highest tertile group (T3)	*P* value
	*n* = 824	(TCBI < 645.6, *n* = 275)	(645.6 ≤ TCBI ≤ 1069.2, *n* = 275)	(TCBI > 1069.2, *n* = 274)	
Age, years	84.0 ± 5.4	84.4 ± 5.6	84.7 ± 5.2	83.0 ± 5.6	**0**.**0006**
Gender, female	564 (68.5%)	200 (72.7%)	181 (65.8%)	183 (66.8%)	0.16
Body weight, kg	51.1 ± 10.3	45.8 ± 8.8	51.3 ± 9.0	56.1 ± 10.4	**<0**.**0001**
BMI, kg/m^2^	22.3 ± 3.5	20.5 ± 3.2	22.3 ± 3.3	24.0 ± 3.8	**<0**.**0001**
NYHA class, III or IV, *n*	425 (51.6%)	160 (58.2%)	140 (50.9%)	125 (45.6%)	**0**.**01**
Logistic EuroSCORE, %	12.8 (9.5, 18.7)	13.9 (10.1, 19.3)	13.3 (10.1, 19.4)	11.7 (8.4, 17.0)	**0**.**04**
EuroSCORE II, %	4.3 (2.7, 7.0)	4.7 (3.1, 7.3)	4.4 (2.7, 7.1)	3.7 (2.4, 6.5)	0.57
STS risk score, %	5.8 (3.9, 8.3)	6.4 (4.7, 8.9)	5.9 (3.9, 8.5)	4.9 (3.4, 7.4)	**0**.**002**
GNRI (*n* = 809)	97.0 ± 9.2	91.8 ± 9.3	97.5 ± 8.5	101.9 ± 9.7	**<0**.**0001**
Comorbidities					
History of heart failure, *n*	237 (28.8%)	92 (33.5%)	81 (29.5%)	64 (23.4%)	**0**.**03**
Hypertension, *n*	648 (78.6%)	212 (77.1%)	213 (77.5%)	223 (81.4%)	0.39
Dyslipidaemia, *n*	477 (57.9%)	143 (52.0%)	167 (60.7%)	167 (61.0%)	0.054
Diabetes mellitus, *n*	201 (24.4%)	58 (21.1%)	72 (26.2%)	71 (25.9%)	0.29
Atrial fibrillation/flutter, *n*	203 (24.6%)	83 (30.2%)	61 (22.2%)	59 (21.5%)	**0**.**03**
History of cancer, *n*	154 (18.7%)	51 (18.6%)	50 (18.2%)	53 (19.3%)	0.94
History of stroke, *n*	91 (11.0%)	36 (13.1%)	28 (10.2%)	27 (9.9%)	0.42
COPD, *n*	92 (11.2%)	38 (13.8%)	31 (11.3%)	23 (8.4%)	0.13
CKD (stage 3 or more), *n*	541 (65.7%)	165 (60.0%)	185 (67.3%)	191 (69.7%)	**0**.**05**
PAD, *n*	136 (16.5%)	46 (16.7%)	46 (16.7%)	44 (16.1%)	0.97
OMI, *n*	54 (6.6%)	19 (6.9%)	17 (6.2%)	18 (6.6%)	0.94
History of coronary revascularization, *n*	186 (22.6%)	64 (23.3%)	65 (23.6%)	57 (20.8%)	0.69
Post-PTAV, *n*	36 (4.4%)	11 (4.0%)	13 (4.7%)	12 (4.4%)	0.92
NT-proBNP, pg/mL	1130 (488, 2876)	1309 (606, 3791)	1154 (465, 2813)	891 (376, 2107)	0.11
Creatinine, mg/dL	0.9 ± 0.4	0.9 ± 0.4	0.9 ± 0.4	1.0 ± 0.4	**0**.**02**
eGFR, mL/min	54.0 ± 18.3	57.0 ± 19.5	53.4 ± 18.2	51.7 ± 17.0	**0**.**003**
Haemoglobin, g/dL	11.6 ± 1.5	11.2 ± 1.5	11.6 ± 1.5	12.0 ± 1.6	**<0**.**0001**
Albumin, g/dL	3.7 ± 0.4	3.6 ± 0.5	3.8 ± 0.4	3.8 ± 0.4	**<0**.**0001**
Total cholesterol, mg/dL	177.6 ± 38.0	158.6 ± 35.2	177.1 ± 33.0	197.2 ± 35.5	**<0**.**0001**
Triglycerides, mg/dL	95.0 (69.0, 129.8)	62.0 (52.0, 74.0)	95.0 (80.0, 108.0)	146.0 (121.8, 183.8)	**<0**.**0001**
Echocardiographic findings					
LVEF, %	60.9 ± 10.7	60.6 ± 11.9	60.8 ± 11.0	61.4 ± 9.0	0.63
AVA, cm^2^	0.67 ± 0.16	0.65 ± 0.16	0.68 ± 0.16	0.69 ± 0.16	**0**.**02**
Peak gradient, mmHg	88.1 ± 30.4	84.7 ± 31.0	88.1 ± 31.5	91.5 ± 28.6	**0**.**04**
Mean gradient, mmHg	50.6 ± 18.7	48.7 ± 19.0	50.9 ± 19.9	52.2 ± 16.9	0.09
AR ≥ moderate, *n*	48 (5.8%)	18 (6.6%)	18 (6.6%)	12 (4.4%)	0.44
MR ≥ moderate, *n*	47 (5.7%)	28 (10.2%)	14 (5.1%)	5 (1.8%)	**<0**.**0001**
TR ≥ moderate, *n*	47 (5.7%)	22 (8.0%)	19 (6.9%)	6 (2.2%)	**0**.**004**
Medications					
Beta-blockers	281 (34.1%)	97 (35.3%)	99 (36.0%)	85 (31.0%)	0.41
ACEI/ARB	456 (55.3%)	143 (52.0%)	160 (58.2%)	153 (55.8%)	0.34
Statins	450 (54.6%)	140 (50.9%)	161 (58.6%)	149 (54.4%)	0.20
Diuretics	389 (47.2%)	154 (56.0%)	128 (46.6%)	107 (39.1%)	**0**.**0003**
Procedural variables					
Procedure time, min	72 (60, 100)	72 (60, 105)	71 (60, 100)	73 (60, 97)	0.25
Fluoroscopy time, min	20 (16, 25)	20 (16, 27)	20 (17, 25)	19 (16, 24)	0.28
Contrast medium volume, mL	63 (46, 96)	63 (45, 95)	61 (47, 100)	63 (45, 95)	0.93
Major complication, *n* (%)	74 (9.0)	28 (10.2)	23 (8.4)	23 (8.4)	0.70
Approach plan					
Local anaesthesia, *n*	486 (59.0%)	158 (57.5%)	151 (54.9%)	177 (64.6%)	0.06
Transfemoral approach, *n*	748 (90.8%)	251 (91.3%)	248 (90.2%)	249 (90.9%)	0.91
Valve size	24.8 ± 2.3	24.7 ± 2.2	25.0 ± 2.3	24.7 ± 2.4	0.28
Valve type					
Edwards SAPIEN-XT, *n*	172 (20.9%)	57 (20.7%)	60 (21.8%)	55 (20.1%)	0.88
Edwards SAPIEN3, *n*	405 (49.2%)	129 (46.9%)	134 (48.7%)	142 (51.8%)	0.51
Medtronic CoreValve, *n*	29 (3.5%)	5 (1.8%)	11 (4.0%)	13 (4.7%)	0.13
Medtronic Evolut R, *n*	142 (17.2%)	52 (18.9%)	43 (15.6%)	47 (17.2%)	0.60
Medtronic Evolut PRO, *n*	64 (7.8%)	29 (10.6%)	23 (8.4%)	12 (4.4%)	**0**.**02**
Boston Scientific LOTUS, *n*	11 (1.3%)	2 (0.7%)	4 (1.5%)	5 (1.8%)	0.50
Balloon expandable, *n*	577 (70.0%)	186 (67.6%)	194 (70.6%)	197 (71.9%)	0.54

*P* values presented in bold indicate that the difference among groups are statistically significant. BMI, body mass index; COPD, chronic obstructive pulmonary disease; CKD, chronic kidney disease; PAD, peripheral artery disease; OMI, old myocardial infarction; Post-PTAV, post-percutaneous transcatheter aortic valvuloplasty; eGFR, estimated glomerular filtration rate; LVEF, left ventricular ejection fraction; AVA, aortic valve area; AR, aortic regurgitation; MR, mitral regurgitation; TR, tricuspid regurgitation; ACEI, angiotensin converting enzyme inhibitors; ARB, angiotensin II receptor blockers.

### Outcomes following transcatheter aortic valve implantation by levels of Triglycerides, total Cholesterol, and Body weight Index

**Figure 1:**
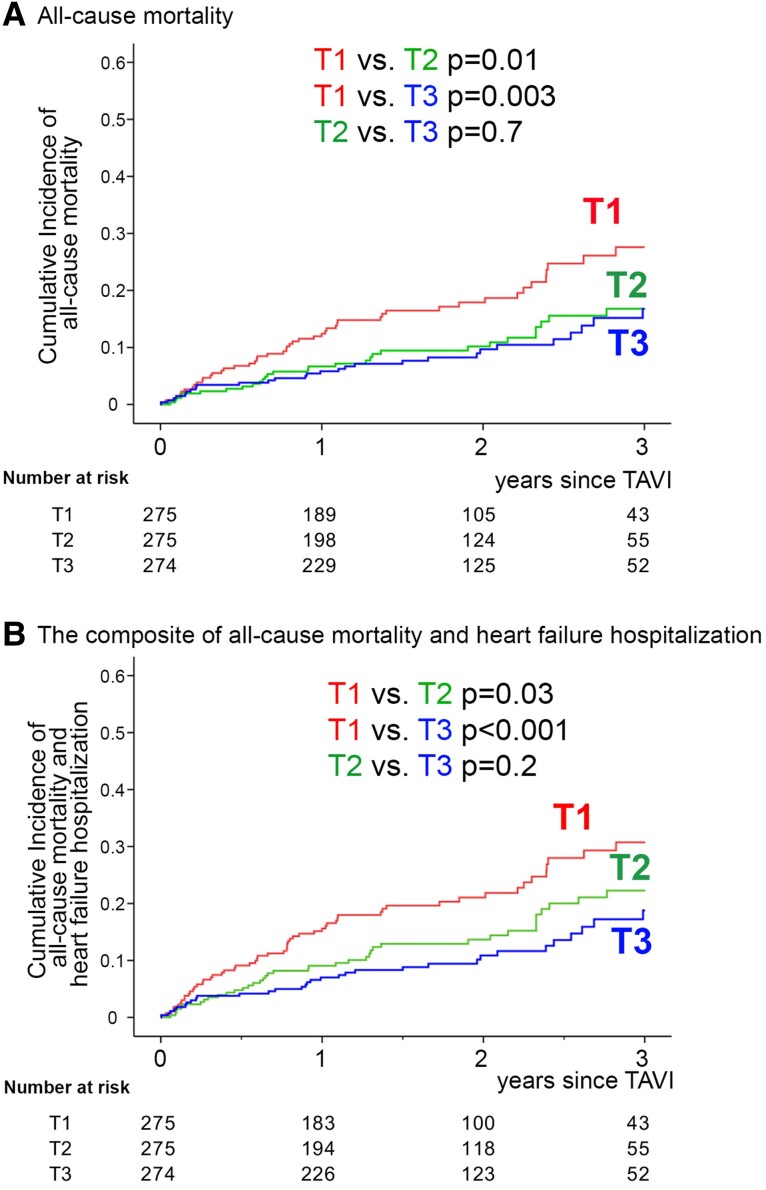
Cumulative incidences of all-cause mortality and the composite of all-cause mortality and heart failure hospitalization by tertile of TCBI. Unadjusted Kaplan–Meier analyses of all-cause mortality (*A*) and the composite of all-cause mortality and heart failure hospitalization (*B*). Patients with lowest, mid, and highest tertiles of TCBI: T1, T2, and T3, respectively.

During the follow-up period of up to 3 years since the TAVI procedure, we identified a total of 106 cases (12.9%) of all-cause death and 128 cases (15.5%) of the composite of all-cause death and heart failure hospitalization. Unadjusted Kaplan–Meier analyses for the primary and secondary outcome measures demonstrated significantly higher cumulative incidences in patients of the T1 group compared to those in the T2 and T3 groups. In contrast, the cumulative incidences of both endpoints were not significantly different between the T2 and T3 groups (*Figure 1A* and *B*).

### Low Triglycerides, total Cholesterol, and Body weight Index as an independent risk factor for all-cause death and the composite of all-cause death and heart failure hospitalization in patients following transcatheter aortic valve implantation

To investigate the prognostic significance of TCBI in patients following TAVI, we conducted Cox proportional hazard analysis using two models to calculate hazard ratios for the primary and secondary outcome measures. Specifically, we assessed low TCBI as a continuous variable (1 SD lower TCBI) and as a numeric variable (lowest tertile TCBI—T1) compared to the other tertiles (T2 and T3).

**Figure 2:**
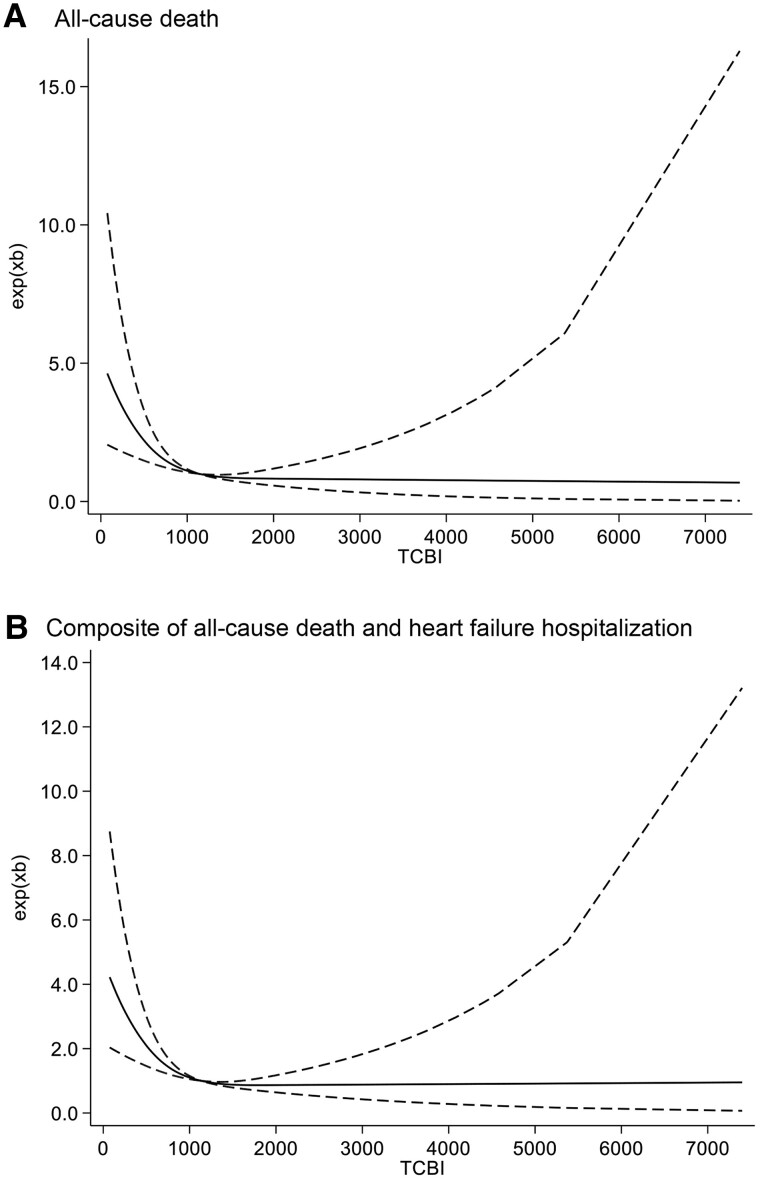
Cubic spline curves for all-cause death and the composite of all-cause death and heart failure hospitalization. Age- and sex-adjusted hazard ratios (black lines) and 95% CI (shaded area) for primary (*A*) and secondary (*B*) outcome measures according to TCBI levels with reference to its median value (834.3).

Our analysis revealed that low TCBI, whether analysed as a continuous or numeric variable, was continuously and significantly associated with an increased risk of both the primary and secondary outcome measures (*Table 2A* and *B*). In the subgroup analysis of patients aged 80 years or older, a 1 SD decrease in TCBI was significantly associated with increased hazard ratios for both the primary outcome and all-cause mortality (*Table 2B*). To explore the differential impact of low TCBI across various surgical risk categories as defined by the STS score, we conducted age- and sex-adjusted multivariate Cox proportional hazards analyses. The results demonstrated that the prognostic significance of low TCBI was more pronounced in the low-risk group (STS < 4) compared to intermediate (STS 4–8) and high-risk (STS > 8) groups (see Supplementary material online, *Table S1*). Furthermore, age- and sex-adjusted cubic spline curve analysis of TCBI as a continuous variable revealed a significant association between TCBI levels lower than 1000 and elevated hazard ratios for both the primary and secondary outcome measures (*Figure 2*). This further supports the notion that lower TCBI is linked to an increased risk of poor clinical outcomes in this patient population.

**Table 2:** Risk of lowest tertile of Triglycerides, total Cholesterol, and Body weight Index for all-cause death and the composite of all-cause death and heart failure hospitalization

	Hazard ratio	95% confidence interval	*P* value
A. Risk of low TCBI as tertile			
Lowest tertile (T1) vs. mid and highest tertile (T2 and T3) TCBI for all-cause death			
Model 1^a^	**2.00**	**1.37–2.94**	**<0**.**001**
Model 2^b^	**1**.**84**	**1.25–2.72**	**0**.**002**
Lowest tertile (T1) vs. mid and highest tertile (T2 and T3) TCBI for the composite of all-cause death and heart failure hospitalization			
Model 1	**1**.**87**	**1.32–2.66**	**<0**.**001**
Model 2	**1**.**68**	**1.17–2.39**	**0**.**004**
B. Risk of low TCBI as continuous variable			
1 SD lower TCBI (a continuous variable) for all-cause death			
Model 1	**1**.**36**	**1.06–1.74**	**0**.**01**
Model 2	**1**.**28**	**1.00–1.64**	**0**.**049**
Model 2 (>80 years old)	**1**.**52**	**1.08–2.13**	**0**.**015**
1 SD lower TCBI for the composite of all-cause death and heart failure hospitalization			
Model 1	**1**.**34**	**1.01–1.68**	**0**.**01**
Model 2	**1**.**27**	**1.01–1.58**	**0**.**037**
Model 2 (>80 years old)	**1**.**44**	**1.08–1.93**	**0**.**014**

Values presented in bold indicate the corresponding results are statistically significant.

^a^Model 1: age, sex, and lowest tertile of TCBI (vs. mid and highest tertiles) or 1 SD lower TCBI.

^b^Model 2: age, sex, diabetes, history of heart failure, chronic kidney disease, atrial fibrillation, and lowest tertile of TCBI (vs. mid and highest tertiles) or 1 SD lower TCBI.

### Slower gait speed and weaker hand grip strength in patients with the lowest tertile of Triglycerides, total Cholesterol, and Body weight Index

**Figure 3:**
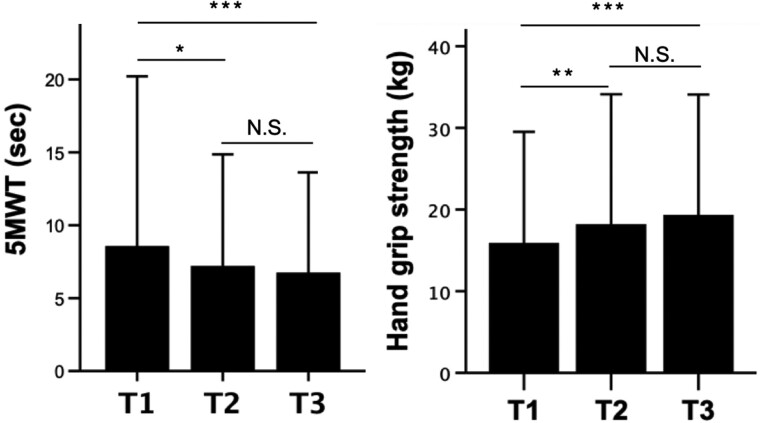
5MWT and handgrip strength according to TCBI tertile. Averages and standard deviations of 5MWT (*A*) and hand grip strength (*B*) by TCBI tertile. ^N.S.^*P* > 0.05, *P < 0.05, **P < 0.01, and ***P < 0.001 by ANOVA, respectively.

To assess potential correlations between TCBI levels and motor functions or frailty in patients following TAVI, we measured and compared the 5MWT and hand grip strength among the three TCBI tertile groups. In the T1 group, the 5MWT results showed significantly slower gait speed, while hand grip strength was notably weaker when compared to patients in the T2 and T3 groups (*Figure 3*). These findings suggest that lower TCBI levels are associated with compromised physical performance and reduced hand grip strength, indicating potential frailty in this subgroup of patients. Consistent with the observed differences, we identified negative and positive modest Spearman’s correlations between TCBI levels and 5MWT and hand grip strength, respectively (see Supplementary material online, *Figure S2*). These correlations further support an association between TCBI and motor functions, underscoring the potential significance of TCBI as a nutritional index related to physical performance and frailty in TAVI patients.

### Higher risk of lower Triglycerides, total Cholesterol, and Body weight Index for all-cause death in patients with serious periprocedural complications of transcatheter aortic valve implantation

To gain further insights into the prognostic significance of TCBI, we conducted separate assessments of the risk associated with the lowest TCBI tertile for all-cause death in patients with and without serious periprocedural complications of TAVI. While no significant difference in the incidence rate of serious periprocedural complications among groups by TCBI. In patients who experienced serious periprocedural complications (*n* = 75), a significantly higher mortality rate during the observational period was noted in the T1 group (12 out of 28 patients, 42.9%), compared to T2 or T3 groups (7 out of 47 patients, 15.2%) (*P* = 0.01). Furthermore, Cox proportional hazard analysis indicated a more significant risk of the lowest TCBI for all-cause mortality in patients with serious periprocedural complications (*Table 3*). These results underscore the importance of considering nutritional status by using TCBI, particularly in patients at high risk for serious periprocedural complications of TAVI. Moreover, given that patients with low TCBI levels are at a very high risk of mortality once they experience serious periprocedural complications of TAVI, the heart team may need to carefully consider procedural strategies, emphasizing safety to avoid complications, in patients with low TCBI.

**Table 3:** Risk of the lowest tertile Triglycerides, total Cholesterol, and Body weight Index in patients with and without major complications of transcatheter aortic valve implantation

	Hazard ratio	95% confidence interval	*P* value
Age- and sex-adjusted risk for all-cause death of the lowest tertile of TCBI			
Without serious periprocedural complications (*n* = 750)	**1.6**	**1.1–2.3**	**0**.**02**
With serious periprocedural complications (*n* = 74)	**4**.**9**	**1.9–12.5**	**<0**.**001**

Values presented in bold indicate the corresponding results are statistically significant.

## Discussion

Our analysis of a prospective multi-centre registry database of patients with severe AS who underwent TAVI revealed compelling insights into the prognostic significance of TCBI. We found that low TCBI levels at the time of the TAVI procedure emerged as an independent risk factor for all-cause mortality and the composite of all-cause mortality and heart failure hospitalization within 3 years. This underscores the importance of considering TCBI as a valuable prognostic marker in patients undergoing TAVI, providing clinicians with a simple and effective tool for risk stratification and patient management. Moreover, our study also sheds light on the association between TCBI and physical function, such as gait speed and reduced muscle strength, both of which are hallmarks of frailty. This finding highlights the potential utility of TCBI not only as a prognostic indicator but also as a valuable marker for assessing overall health status and functional capacity in TAVI patients. Additionally, this study revealed that the negative prognostic impact of low TCBI was further amplified in patients with age of 80 or older and those who with low procedural risk for TAVI. Finally, patients who encountered serious periprocedural complications during TAVI faced a significantly higher risk of all-cause mortality when they had lower TCBI levels. These findings emphasize the importance of paying close attention to TCBI, particularly in older patients and those at high risk for procedural complications, to optimize patient outcomes and inform clinical decision-making.

The indication for TAVI has expanded to include patients at lower surgical risk, making it a less invasive treatment option for severe AS. However, the majority of patients undergoing TAVI remain elderly, as the severity of AS is often associated with aging.^17^ In our study population within the LAPLACE-TAVI registry, more than 80% of patients were over 80 years old. Malnutrition or poor nutritional status has been recognized as a significant prognostic factor in elderly patients undergoing surgical and transcatheter aortic valve replacement.^1,6^ Various methodologies have been used to quantify nutritional status in these patients, including questionnaire-based assessments, such as the mini nutritional assessment-short form,^1^ malnutrition universal screening tool, and short nutritional assessment questionnaire,^18^ as well as objective parameters-based indices like GNRI,^19^ CONUT score, and prognostic nutritional index.^6^ These conventional nutritional indices have shown promise in risk stratification for TAVI patients, but may be complex and time-consuming to calculate, limiting their widespread clinical use. In contrast, TCBI overcomes the challenges associated with conventional indices. By simply multiplying three routinely measured objective parameters—serum triglycerides, serum total cholesterol, and body weight—TCBI provides a straightforward and easily calculable index. Numerous studies have demonstrated the validity of TCBI as a nutritional index and prognostic indicator in various cardiovascular disease contexts, including CAD,^7,11^ heart failure,^9^ critical patients requiring circulatory assist devices,^8^ stroke,^10^ and even patients after TAVI.^20^ Similarly, the present study confirms that low TCBI, both as a numeric and continuous variable, is significantly associated with an increased risk of all-cause mortality following TAVI over a 3-year period. Importantly, the prognostic impact of TCBI remains independent of traditional procedural risk scores, such as the EURO II score, suggesting its potential value in further refining risk stratification for TAVI patients. Moreover, a subgroup analysis indicated that the adverse prognostic impact of low TCBI was more pronounced in patients with lower procedural risk indicated by STS score < 4. These findings suggest that TCBI may be particularly useful for risk stratification in lower-risk patients undergoing TAVI.

Despite technical advancements in transcatheter valve systems, increased operator experience, and technical refinements, concerns regarding life-threatening periprocedural complications remain one of the most significant issues of TAVI.^21^ Although the overall incidence of serious complications is decreasing over time,^22^ the short-term and long-term mortality rates in cases that encountered these are still very high, ranging from 6.4–14.1% within 30 days after the TAVI procedure.^23^ Accordingly, the indication of TAVI and its procedural risk should be further evaluated very carefully in cases who are vulnerable to serious periprocedural complications. In the present study, serious periprocedural complications doubled the 1-year mortality rate in patients following TAVI, respectively. Moreover, while no significant effect of TCBI on the occurrence of serious periprocedural complications was observed, the prognostic impact of low TCBI was substantially enhanced in patients with complications of TAVI. These novel findings, which were not shown in a previous study assessing TCBI as a prognostic marker in patients after TAVI,^20^ indicate that, in patients with low TCBI, the heart team should select procedural strategies that place an emphasis on safety with respect to approach sites, and valve type and size. By tailoring procedural decisions to individual patients, with special attention given to those with low TCBI, we can potentially reduce the occurrence of serious complications and improve overall patient outcomes. Further research and collaborative efforts in risk assessment and patient selection will be crucial in refining TAVI procedures and maximizing the benefits for this vulnerable patient population.

Frailty is a prevalent condition in elderly individuals with severe AS, and its prognostic impact on poor outcomes following TAVI has been demonstrated.^24^ As a result, for determining the indication of TAVI and assessing procedural and prognostic risks, particularly in elderly patients, the VARC-2 consensus has recommended frailty assessment before the TAVI procedure,^24^ which includes 5MWT and hand grip strength as two of the three indicative parameters of frailty in patients undergoing SAVR and TAVI. Moreover, previous studies have independently demonstrated associations between increased 5MWT^25^ or decreased hand grip strength^26^ and poor outcomes following TAVI. In the present study, we found that 5MWT was significantly higher in the group with the T1 compared to the T2 and T3 groups, and there was a modest bivariate correlation between TCBI and 5MWT. Similarly, hand grip strength was significantly lower in the T1 group compared to the other two groups. These findings indicate that the extent of frailty was particularly pronounced in patients with low TCBI. Moreover, when considering the significantly poorer outcomes of patients in the lowest TCBI tertile, our findings suggest a possible link among poor nutrition, higher frailty, and worse outcomes in patients with severe AS who undergo TAVI, which was not indicated in a previous study.^20^

### Limitations

The present study has several limitations that should be considered when interpreting the results. First, the retrospective nature of the multi-centre registry-based cohort study may limit the ability to establish causality, despite the prospective data collection in the LAPLACE-TAVI registry database. Although this study employed multiple Cox models to adjust for potential confounding factors, unmeasured or residual confounders may still influence the observed associations. Second, it is important to acknowledge that the vast majority of participants in the LAPLACE-TAVI registry were of Japanese ethnicity. As such, the generalizability of the present findings to other ethnic populations should be interpreted with caution. Third, the relatively small number of participants and endpoint incidence in the present analysis may have limited the statistical power and precision of the results. Specifically, participants in this study represent a subset of the entire LAPLACE-TAVI registry due to the availability of triglyceride data, which was not mandatory data field within the registry. Consequently, over 200 cases were excluded from the analysis, potentially affecting the generalizability of our findings. Larger sample sizes and longer follow-up periods may be needed to further confirm the robustness of the association between TCBI and outcomes following TAVI. Fourth, as TCBI data were available only at a single time point in this study, the prognostic impact of temporal changes in TCBI at multiple time points remains to be explored. Longitudinal assessments of TCBI and its association with outcomes over time could provide valuable insights into the potential use of TCBI not only as a biomarker but also as a therapeutic target in the management of TAVI patients. Fifth, the intensity of statin therapy was not available in the LAPLACE-TAVI registry, which may influence lipid profiles and the calculated TCBI. This absence of data may limit our ability to assess the potential impact of statin therapy intensity on the prognostic value of TCBI.

Despite these limitations, our study demonstrated that reduced TCBI was independently associated with an increased risk of poor outcomes following TAVI. Given that elderly patients undergoing TAVI are inherently at high risk, the prognostic significance of TCBI may be even more substantial in this population compared to previously investigated cohorts. Therefore, TCBI holds promise as a valuable prognostic indicator in TAVI patients, highlighting the need for further research to better understand its clinical utility and potential implications for patient care and outcomes in this vulnerable population.

## Conclusions

Our study adds insights into risk stratification and prognostication in TAVI patients, suggesting that TCBI could serve as a useful tool for identifying high-risk individuals who may benefit from tailored procedural strategies and enhanced post-procedural care. The evidence presented in this study supports the potential value of TCBI as a nutritional and prognostic marker in patients undergoing TAVI, particularly in the elderly. Moreover, the potential effects of nutritional interventions aimed at raising the level of TCBI for better outcomes in patients following TAVI may need to be evaluated in future studies. Incorporating TCBI assessment into the preoperative evaluation may contribute to more informed decision-making and ultimately improve patient outcomes in this high-risk population.

## Supplementary Material

oeaf008_Supplementary_Data

## Data Availability

The data underlying this article will be shared on reasonable request to the corresponding author.

## Lead author biography

Dr Shinichiro Doi is an assistant professor of the Department of Cardiovascular Biology and Medicine at the Graduate School of Medicine, Juntendo University in Tokyo, Japan. His clinical area of expertise is interventional cardiology, specifically catheter interventions for structural heart disease. His research centres on various risks for poor outcomes following catheter intervention, such as malnutrition in patients undergoing percutaneous coronary intervention (PCI) and transcatheter aortic valve implantation (TAVI). He also plays a leading role in the multicentre TAVI registry in Japan, the LAPLACE-TAVI registry.
