# Supplementary material for: The Triglycerides, total Cholesterol, and Body weight Index associating with frailty and predicting poor outcome after transcatheter aortic valve implantation: insights from LAPLACE-TAVI registry
Source: Eur Heart J Open. 2025 Jan 28;5(1):oeaf008. doi: 10.1093/ehjopen/oeaf008 (PMC11848272; doi:10.1093/ehjopen/oeaf008)
Supplement: oeaf008_Supplementary_Data [file oeaf008_supplementary_data.pdf]

Supplementary materials

Figure S1: A flow diagram of the study

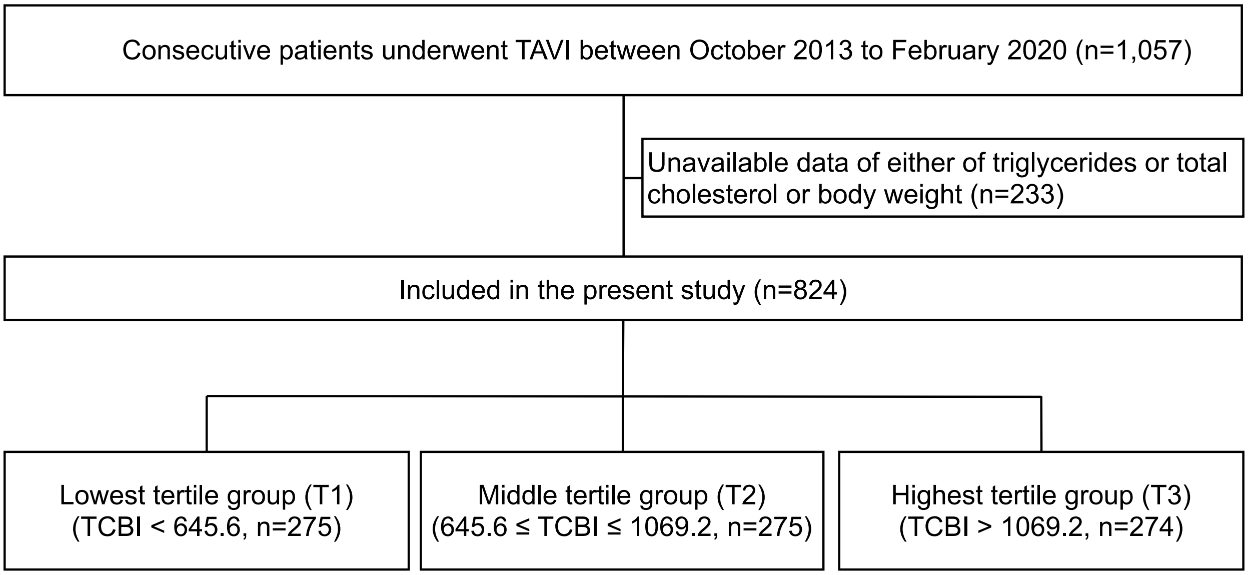

Figure S2: Correlations between TCBI and 5MWT or hand-grip strength

A Correlation between TCBI and 5MWT (Spearman's correlation coefficient (r): -0.226, p<0.001)

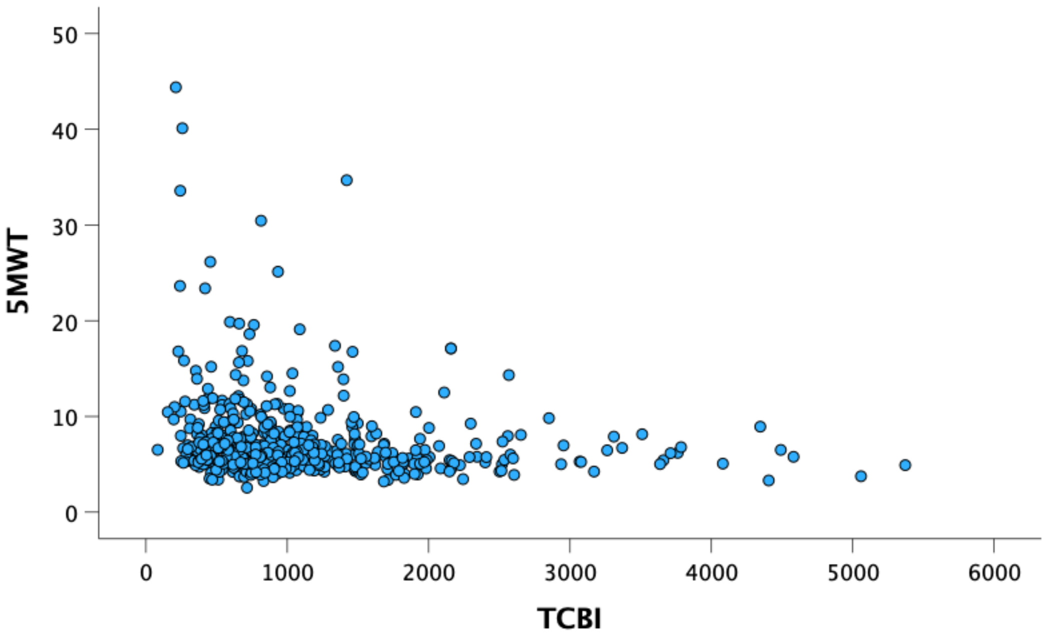

B Correlation between TCBI and hand grip strength (r= 0.229, p<0.001)

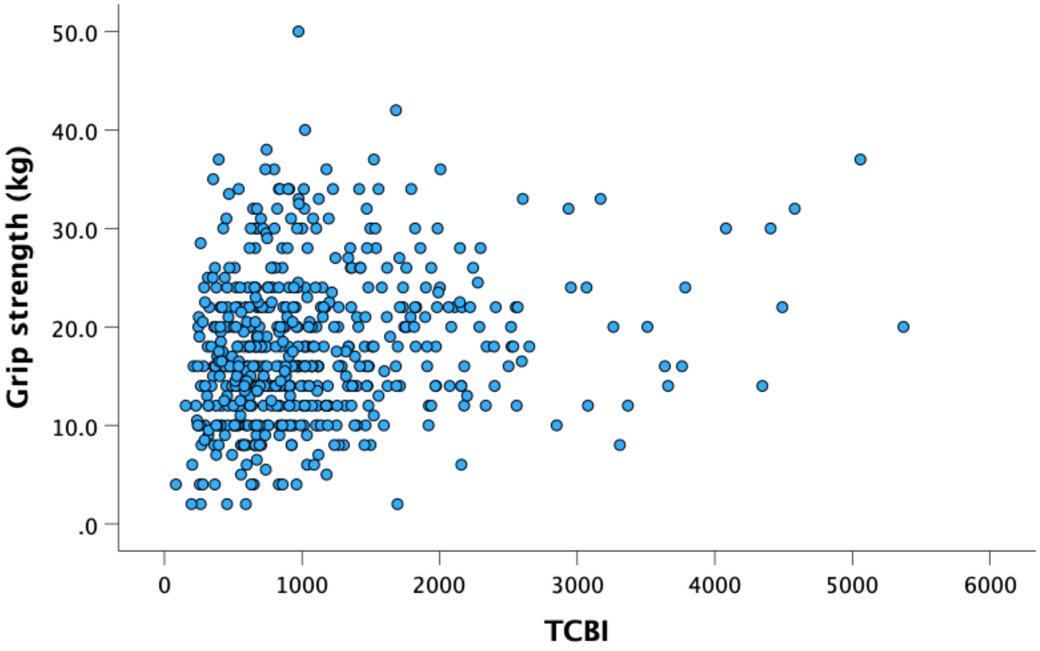

Table S1: **Age- and Sex-Adjusted Impact of Low TCBI on Outcomes by a Procedural Risk Category (STS Score)**

Risk category by STS score	Hazard Ratio	95% confidence interval	p-value
Low risk (STS: <4)	2.10	0.98-5.18	0.048
Mid risk (STS: 4-8)	1.71	1.05-2.80	0.032
High risk (STS:>8)	1.16	0.73-1.86	0.53
